# Ten2Twenty-Ghana: a randomised controlled trial on the efficacy of multiple micronutrient-fortified biscuits on the micronutrient status of adolescent girls

**DOI:** 10.1017/S0007114523002234

**Published:** 2024-02-28

**Authors:** Fusta Azupogo, Abdul-Razak Abizari, Edith J. M. Feskens, Hans Verhoef, Inge D. Brouwer

**Affiliations:** 1 Department of Family and Consumer Sciences, Faculty of Agriculture, Food and Consumer Sciences, University for Development Studies, Tamale, 1882, Ghana; 2 Institute for Global Nutrition, University of California, Davis, USA; 3 Formerly of the Department of Nutritional Sciences, School of Allied Health Sciences, University for Development Studies, Tamale, Ghana; 4 Division of Human Nutrition and Health, Wageningen University and Research, Wageningen, The Netherlands

**Keywords:** Adolescent girls, Menarche, Fortified food, Anaemia, Micronutrient status, Ghana

## Abstract

Adolescent girls are an important target group for micronutrient interventions particularly in Sub-Saharan Africa where adolescent pregnancy and micronutrient deficiencies are common. When consumed in sufficient amounts and at levels appropriate for the population, fortified foods may be a useful strategy for this group, but little is known about their effectiveness and timing (regarding menarche), particularly in resource-poor environments. We evaluated the effect of consuming multiple micronutrient-fortified biscuits (MMB), sold in the Ghanaian market, 5 d/week for 26 weeks compared with unfortified biscuits (UB) on the micronutrient status of female adolescents. We also explored to what extent the intervention effect varied before or after menarche. Ten2Twenty-Ghana was a 26-week double-blind, randomised controlled trial among adolescent girls aged 10–17 years (*n* 621) in the Mion District, Ghana. Biomarkers of micronutrient status included concentrations of Hb, plasma ferritin (PF), soluble transferrin receptor (TfR) and retinol-binding protein (RBP), including body-iron stores. Intention-to-treat analysis was supplemented by protocol-specific analysis. We found no effect of the intervention on PF, TfR and RBP. MMB consumption did not affect anaemia and micronutrient deficiencies at the population level. MMB consumption increased the prevalence of vitamin A deficiency by 6·2 % (95 % CI (0·7, 11·6)) among pre-menarche girls when adjusted for baseline micronutrient status, age and height-for-age *Z*-score, but it decreased the prevalence of deficient/low vitamin A status by −9·6 % (95 % CI (−18·9, −0·3)) among post-menarche girls. Consuming MMB available in the market did not increase iron status in our study, but reduced the prevalence of deficient/low vitamin A status in post-menarcheal girls.

Adolescent girls are an important target group for micronutrient interventions, especially in Sub-Saharan Africa where micronutrient deficiencies and adolescent pregnancy are prevalent. Globally, West and Central Africa have the highest adolescent birth rates (115 births per 1000 girls)^(1)^. In Ghana, a third of girls are married by 18 years^(2)^, and 14 % of girls aged 15–19 years have ever given birth. In comparison with urban and southern Ghana, rural and northern Ghana have a strikingly higher rate of teenage marriage and pregnancy^(3)^. Addressing the nutritional needs of this vulnerable group is critical to prevent complications related to anaemia and to ensure healthy maternal and infant outcomes during pregnancy^(4–6)^.

In Ghana, one-fifth of adolescent girls aged 15–19 years have iron deficiency (ID) and about 14·5 % are estimated to have iron deficiency anaemia (IDA)^(7)^. A recent study in the Northern and Volta regions of Ghana found that a quarter of school-going adolescent girls are anaemic^(8)^. The burden of anaemia and IDA is substantially higher for adolescent girls due to the increased demand for puberty, menstrual losses and dietary inadequacies, particularly in low socio-economic settings^(5,9)^. The Ghana micronutrient survey estimates that less than 1 % of adolescent girls aged 15–19 years have vitamin A deficiency (VAD)^(7)^ but a study^(10)^ in the Ashanti region of Ghana found one-third of girls aged about 15 years with VAD, suggesting that the prevalence rates may also be context and age-specific.

Iron absorption must balance iron losses to sustain the physiological process, particularly erythropoiesis^(11)^. According to Hallberg *et al.*^(12)^, the average menstrual blood loss in 15-year-old girls is ∼28 mL per period, corresponding to a daily loss of ∼0·4 mg iron. Research on the interrelations between menarche and iron status is inconclusive. Menstruation-induced blood loss may *increase* iron absorption through the homeostatic mechanism of up-regulation during a deficiency^(13)^. One study suggests that poor iron status reduces hepatic hepcidin synthesis, and low circulating hepcidin increases dietary iron absorption^(14)^; this may benefit post-menarche girls through the up-regulation mechanism earlier mentioned. On the other hand, menstruation-related inflammation would result in *reduced* iron absorption^(9)^. It is therefore unclear which segment of this population (whether pre- or post-menarche) would benefit most from a dietary intervention given the differing dynamics of blood loss and iron absorption. Furthermore, the demand and use of vitamin A increases during the pre-ovulatory phase to produce reproductive cells and endometrium, and during the post-ovulatory period to maintain the endometrial layer^(15)^, which suggests that post-menarche girls may benefit more from a vitamin A intervention.

School-based interventions have the potential to improve adolescent girls’ nutrition and health^(16)^, breaking the intergenerational cycles of malnutrition and deprivation. Multiple micronutrient-fortified foods (MMF), such as multiple micronutrient-fortified biscuits (MMB), can be a more practical and effective strategy for addressing micronutrient deficiencies of teenage females in low socio-economic environments than supplements and fortified flours^(17)^. Biscuits are convenient and relatively easy to manage and distribute, and they have a long shelf life^(18)^. Also, biscuits are snacks rather than meals, are unlikely to replace home-cooked meals and are well-accepted by the adolescent population^(18,19)^. However, data are scarce on the impact of MMF on adolescent girls’ micronutrient status in Sub-Saharan Africa; the information available is mostly from high-income countries^(20,21)^. We conducted a 26-week randomised controlled trial (RCT) to assess the effect of a 5-d weekly consumption of MMB, a product available in the Ghanaian market, compared with unfortified biscuits (UB), on the micronutrient status of adolescent girls. We also explored to what extent the intervention effect varied before or after menarche.

## Methodology

### Study area and participants

The study population consisted of pre-and post-menarche adolescent girls aged 10–17 years in 14 communities in the Mion District, the Northern Region of Ghana. The district is mainly rural (about 91 %), and our previous secondary analysis of data^(22)^ suggests a high prevalence (64·6 %) of anaemia among adolescent girls in the rural northern savannah agroecological zone.

### Study design

Ten2Twenty-Ghana was a 26-week follow-up double-blind, RCT. The study design details, including the inclusion and exclusion criteria, have previously been described in detail elsewhere^(23)^. A non-targeted approach, including both anaemic and non-anaemic girls, was used to randomise girls within strata defined by menarche status (pre-and post-menarche) into two parallel treatment arms receiving nutrition/health education (5 different occasions) with either a 5-d weekly MMB or UB for 26 weeks. This study was conducted according to the guidelines laid down in the Declaration of Helsinki and all procedures involving human subjects/patients were approved by the Navrongo Health Research Centre Institutional Review Board (NHRCIRB323). The Ghana Education Service granted written approval, and leaders of participating communities provided informed consent. Participation was entirely voluntary, and the girl gave her assent, after receiving signed/thumb-printed informed consent from her guardian or parent. The RCT was also registered prospectively with the Netherlands Clinical Trials Register https://onderzoekmetmensen.nl/en/trial/26929 with registration number NL7487.

### Sample size

The sample size was estimated based on 80 % power, a one-sided hypothesis and a 5 % level of significance for three variables – Hb, serum ferritin and serum retinol. For both anaemic and non-anaemic girls in this sample, the sd for Hb was 12·9 g/l, while for solely anaemic girls, it was 8·4 g/l^(22)^. Therefore, 141 girls per group for a non-targeted strategy and 122 girls per group for just only anaemic girls were needed to detect a minimum difference in mean Hb of 3·8 g/l between the MMB and UB groups. Based on a previous study’s sd for serum ferritin of 20·1 µg/l^(24)^, 57 girls per group were needed to find a mean difference of 9·5 µg/l in ferritin between the MMB and UB groups. Last but not the least, using a prior study’s^(24)^ sd for serum retinol (0·3 µmol/l), 23 females per group were needed to find a mean difference of 0·22 µmol/l in serum retinol between the MMB and UB groups. The expected mean differences for Hb (3·8 g/l), serum ferritin (9·4 µg/l) and serum retinol (0·11 µmol/l) were biologically plausible^(25)^. We took into account the bigger estimate (*n* 141) of the three variables (Hb, serum ferritin and retinol) and a minimum sample of 155 girls per group used, considering a maximum attrition rate of 10 % during follow-up. The study comprised a total of 4 groups with pre-menarche and post-menarche girls (310 pre-menarche and 310 post-menarche) randomly assigned into the parallel arms of the RCT; thus, 620 adolescent girls were needed.

### Sampling procedure

**Fig. 1.**
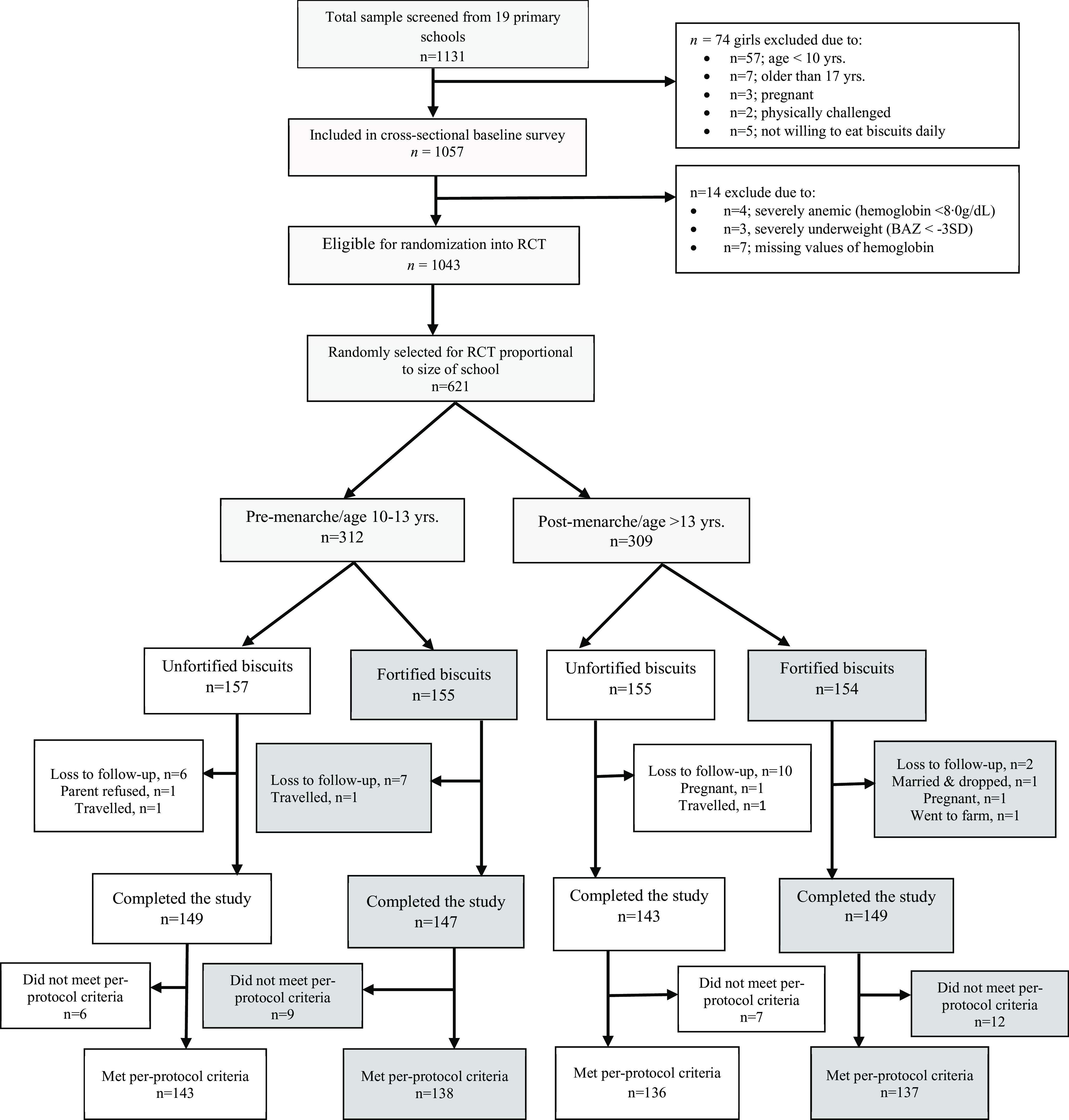
The flow of participants, as per CONSORT guidelines including reasons for non-enrolment and drop-out in the RCT.

In a survey conducted in November/December 2018, we invited and recruited 621 girls who met the selection criteria (online Supplementary Table S1) out of a total of 1057 adolescent girls. Subjects were recruited for the RCT in February 2019 (during the dry season), and treatment and follow-up finished in September 2019, during the rainy season’s peak. Only primary school girls were included in the study. Menarche status was based on recall at screening and the age of the girl (> 13 years) using the average age of menarche in Ghana from the literature^(26)^. The girls were randomly assigned to the intervention and control groups using a two-stage sampling technique with probability proportional to school and menarche group size. Firstly, we created and sorted random numbers (between 0–1) by menarche group within schools in ascending order (lowest to highest). Girls from each school’s menarche group were then enrolled by the project coordinator until the sample size requirement for the menarche group was met. For selected girls in each menarche group and school, a second set of random numbers between 0 and 1 was created, and girls with random numbers less than 0·5 were assigned to the UB group, while those with random numbers greater than or equal to 0·5 were assigned to the MMB group. Fig. 1 depicts the flow of participants in the RCT, including reasons for non-enrolment and drop-out.

### Treatment and control

Girls in the treatment arm of the RCT received the Obaasima MMB, enriched with 11 vitamins (vitamins B_1_, B_2_, B_6_, B_12_, A, D, K_1_, E, niacin, folic acid and ascorbic acid) and 7 minerals (Zn, Ca, Fe, Cu, I, Se and Mg) (Table 1). Obaasima is a partnership project between the German Development Cooperation and the private sector in Ghana to develop ‘*Affordable and Nutritious Foods for Women (ANF4W)*’. Obaasima products are fortified to fulfil 15 % and 30 % of the recommended dietary allowance of the fortified minerals and vitamins respectively, for young women aged 19–30 years (Sight and Life; personal communication). Ferrous fumarate (8·2 mg/100 g) and dry vitamin A palmitate (1·0 mg/100 g) were the fortificants for iron and vitamin A respectively. Girls in the control group consumed biscuits similar in calories and appearance to the MMB. As a result of the national policy mandating wheat flour fortification (with vitamin A, folic acid, vitamin B_12_, thiamine, riboflavin, niacin, iron and zinc), the UB also contained a limited amount of micronutrients (Table 1).

**Table 1.** Nutrient content of biscuits for Ten2Twenty-Ghana RCT

No.	Nutrient	Product name	Nutrient content of fortified biscuits (mg) per serving (51·3 g)	^*^Nutrient content (mg) of unfortified biscuits per serving (51·3 g)
1	Vitamin A	Dry vitamin A palmitate	0·504	0·10
2	Vitamin D	Dry vitamin D_3_	0·005	0·00
3	Vitamin E	Dry vitamin E	6·00	0·00
4	Vitamin K	Dry vitamin K_1_	0·05	0·00
5	Thiamine	Thiamine mononitrate	1·20	0·43
6	Riboflavin	Riboflavin	1·20	0·23
7	Niacin	Niacinamide	14·00	3·03
8	Vitamin B_6_	Pyridoxine Hydrochloride	1·60	0·00
9	Folic acid	Folic acid	0·311	0·11
10	Vitamin B_12_	Vitamin B_12_	0·002	0·001
11	Ascorbic acid	Ascorbic acid	70·00	0·00
12	Calcium	Calcium carbonate	150	0·00
13	Cupper	Cupper Gluconate	0·20	0·00
14	Iodine	Potassium Iodide	0·04	0·00
15	Iron	Ferrous Fumarate	4·05	1·03
16	Magnesium	Magnesium oxide	52·50	0·00
17	Selenium	Sodium Selenite	0·012	0·00
18	Zinc	Zinc Oxide	2·38	1·45

* Obtained from the lab division of Mass Industries, Tema-Ghana; the nutrient content reflects the fortification level of wheat flour in Ghana as by law.

The girls consumed a pack of biscuits (51·3 ± 3·2 g) *ad libitum* as a snack on each school day (Monday through to Friday), for 26 weeks, in the teacher and/or field assistant’s presence. A pack of the MMB or UB contained between 8–10 pieces of biscuits, with the average weight of a piece being 5·6 ± 0·5 g. The whole pack of biscuits had to be consumed at each feeding session but leftovers in pieces (if any) were recorded with a daily case report form as pieces; the case report also captured subjects’ attendance at feeding sessions, adverse events and severe adverse events during the intervention. The recording was done by the schoolteacher who supervised the feeding. All schoolteachers who supervised the feeding received a 1-d training on completing the case report form and participated in a 5-d pre-trial run-in practice of the feeding arrangement and case report filling. The teachers were in turn supervised by trained field assistants twice weekly and the lead author weekly. The pre-trial run-in biscuits were procured from the open market and were similar in nutrient content to the UB and comparable in size (50 g) to both the MMB and UB. The research team and subjects were blinded in our investigation by repackaging the biscuits into clear zip-lock bags with yellow and red tags^(23)^.

### Plasmodium infection and deworming

We screened for current or recent *Plasmodium* infection at baseline, mid-point (thirteenth week) and endline with malaria rapid diagnostic test (First Response; Premier Medical, Somerset, New Jersey, USA). To evaluate the accuracy of the malaria rapid test kits, we conducted malaria microscopy on approximately 11 % (68 out of 621) of subjects, during the mid-point malaria screening. Malaria rapid diagnostic test uses histidine-rich protein-2, which is specific for *P. falciparum*^(27)^ *; P. falciparum* accounts for at least 75 % of *Plasmodium* infections in northern Ghana^(28,29)^. Girls who tested positive for *Plasmodium* infection at any time point were given artemether-lumefantrine (80 mg/480 mg twice daily for 3 d) as treatment^(30)^. Similarly, girls who reported fever and/or headache during the intervention were also tested and treated for *Plasmodium* infection when positive. Finally, at baseline, all subjects were dewormed with a single dose of mebendazole 400 mg chewable tablets.

### Biochemical measurements and analysis

We collected non-fasting venous blood at baseline and 26 weeks after the start of intervention into Na-Heparin Vacutainers (Becton-Dickinson Diagnostics) for the measurement of plasma concentrations of plasma ferritin (PF), soluble transferrin receptor (TfR), retinol-binding protein (RBP) as retinol equivalents (µmol/l) and inflammation biomarkers: plasma concentrations of C-reactive protein (CRP) and *α*-1-acid glycoprotein (AGP). The conversion from mg/l to µmol/l was 15 mg/l RBP is equal to 0·7 µmol/l RBP. We also assessed Hb, with the HemoCue 301 photometer (Ängelholm, Sweden; 0·1 g/dl precision) by finger prick at baseline and using venous blood at the endline. The VitMin Lab (Willstätt, Germany) measured PF, TfR, RBP, CRP and AGP using a combined sandwich ELISA^(31)^. All measurements were duplicated and were repeated where the CV (inter-assay) was >10 %. The CVs for the various indicators were PF, 2·3 %; TfR, 3·6 %; RBP, 3·6 %; CRP, 5·8 %; and AGP, 8·1 %. Certified quality control samples from the CDC/Atlanta and Bio-Rad Liquichek controls (Bio-Rad) were used to produce calibration curves.

### Anthropometry

In the baseline survey, we measured height and weight in duplicates to the nearest 0·1 decimal with the Seca stadiometer and digital weighing scale, respectively, following standard procedures^(32)^. The average of the duplicate measurements was used in the analysis.

### Covariates

We included information on several covariates collected during face-to-face interviews using a pre-tested questionnaire. The child-level covariates included age, ethnicity and religion, and the girls’ dietary diversity score from a single qualitative 24-h dietary recall, based on ten food groups^(33)^. The dietary data also included the frequency of consuming different food groups in the last month, including animal source foods (eggs, fish, meat, dairy products), legumes/nuts/seeds, vitamin A-rich dark green leafy vegetables and other vitamin A-rich fruits and vegetables. Maternal-level covariates included the mother’s age, literacy, education and work status, as well as an index of the mother’s household decision-making participation based on the 8-item final decision-making index of the demographic and health survey as used previously^(34)^. Household-level covariates included paternal literacy, education and work status as well as a household roster to compute the household size and ratio variables for dependency, literacy and female-to-male ratio. Households were classified as food secure or food insecure based on the Food Insecurity Experience Scale^(35)^. Lastly, we created a household asset index using principal component analysis^(36)^ and then ranked the subjects’ households into quintiles of household wealth. The covariates are described in detail in the supplementary material file.

### Adjustment of micronutrient biomarkers and definitions

We adjusted the micronutrient biomarkers (PF, TfR and RBP) for inflammatory biomarkers (the concentration of CRP and AGP) on a continuous scale and *Plasmodium* infection (as a dichotomous variable) using the BRINDA group’s internal regression correction approach^(37)^. The details of the micronutrient biomarker adjustment are presented in the supplementary material. Anaemia and the severity of anaemia were defined according to WHO criteria^(38)^. CRP > 5 mg/l and/or AGP > 1·0 g/l^(39)^ was used to indicate sub-clinical inflammation (SCI). We defined ID using the adjusted biomarkers as (1) PF < 15µg/l^(39)^; (2) TfR > 8·3 mg/l^(31)^; (3) PF < 15 µg/l or TfR > 8·3 mg/l; and (4) finally with unadjusted PF as PF < 15 µg/l for girls without SCI but PF < 70 µg/l for girls with SCI^(39)^. IDA was defined as concurrent anaemia and ID. VAD was defined as RBP < 0·7 µmol/l, while low/marginal vitamin A status was defined as RBP ≥ 0·7 but <1·05 µmol/l^(40)^ after adjusting RBP for inflammation. Hb was not adjusted for altitude as no adjustment is needed for populations living below 1000 m above sea level^(38)^ or smoking status which was not recorded in our population. Being stunted was defined as a height-for-age *Z*-score (HAZ) < -2 SD and BMI-for-age *Z*-score (BAZ) was categorised into thinness (BAZ < -2 SD), normal weight (-2 SD ≤ BAZ ≤ +1 sd), overweight/obesity (BAZ ≥ +1 SD)^(41)^. Treatment adherence was defined with the percentage of the total amount (gram) of biscuits each girl consumed, considering the total amount that was scheduled to be consumed for the 26 weeks of intervention. We computed body iron stores using Cook’s formula^(42)^, using the adjusted ferritin and transferrin receptor concentration.

### Statistical analysis

We computed HAZ and BAZ using WHO AnthroPlus with WHO 2007 growth reference for 10–19 years girls. Data analysis was conducted with SAS 9.4 (SAS Institute Inc.) and STATA (StataCorp., v.13.0). The primary analysis of the treatment effect was intention-to-treat analysis with multiple imputations of missing endline outcome variables (*n* 33). The chained-equations method, which presumes that the data are missing at random^(43)^ was used with the PROC MI SAS command. Aside from the school, all prediction equations in the multiple imputations incorporated covariates at the girl, maternal and household levels.

The outcome variables were Hb, body iron stores and the adjusted log-transformed PF, TfR and RBP. We estimated the differences in post-intervention measurements between the two groups with adjustment for baseline values of each measurement as in an ANCOVA, using a linear mixed model (Proc Mixed) in SAS. In the analysis, the school was included as a random intercept. We did not transform Hb and body iron stores, which were normally distributed. Adjustments for covariates did not influence our results and were subsequently dropped.

We estimated the post-intervention prevalence differences^(44)^ in micronutrient deficiencies between MMB and UB groups with the post-estimation command, *adjrr* after running logit models on each micronutrient problem in STATA; the approach automatically adjusts for complex survey design. Two statistical models were created; model 1 (crude model) included the biscuits group and the study design effect (menarche status at enrolment). Model 2 adjusted for the girl’s baseline micronutrient biomarkers (Hb, PF, TfR and RBP), age and HAZ. To explore to what extent the magnitude of intervention effects depended on menarche status, we added an interaction term of biscuits and menarche status at enrolment (Biscuits*menarche status) and as well produced stratified estimates by menarche status.

### Sensitivity and subgroup analyses

We first conducted a per-protocol analysis, restricted to girls with adherence ≥ 80 %. We also conducted a subgroup analysis for subjects who were anaemic at baseline, ensuring that the intervention’s effect is plausibly not masked by tissue saturation of nutrient-replete girls.

## Results

### Baseline characteristics

Of 621 girls randomised, 588 (94·7 %) completed the study, 94·5 % in the MMB group and 94·9 % in the UB group (Fig. 1). Girls who dropped out of the study (*n* 33) had higher CRP, BAZ and earlier menarche at baseline than those who completed the study. Treatment adherence was 90·3 % and 88·1 % in the MMB and UB respectively. The girls’ mean age at baseline was 12·8 ± 2·0 years (Table 2). The mean HAZ and BAZ were −0·10 ± 1·2 and −0·7 ± 0·9, respectively. Overall, about 17·4 % of the girls were stunted, 7·1 % were underweight, and less than 2 % were overweight/obese at baseline. A little over 40 % of the girls had SCI at baseline, mainly in the late convalescence phase (high AGP and normal CRP). Although inflammation did not differ by menarche status, pre-menarche girls were more likely than post-menarche girls to have *Plasmodium* infection at the mid-point (22·1 % *v*. 13·9 %, *P* = 0·008). Further, about three-fifths of subjects with post-intervention anaemia also had SCI at either time point. About a fifth of the subjects reported an adverse event during the intervention with more than half of reported adverse events being a fever/malaria. In our study, about 40 % of the girls were anaemic; a little over half were iron deficient and close to a quarter had IDA at baseline. The prevalence of ID was about five times higher when TfR was used instead of PF (50 % *v*. 11 %). About one-third of the girls were either vitamin A deficient or had a marginal vitamin A status. After 26 weeks of intervention, 37 (6·0 %) of the 312 pre-menarche girls attained their menarche. The girls were from low-income households, with just 10 % of their mothers being literate and only a fifth of the households having access to food (Table 3). Approximately 90 % of the mothers were over the age of 50.

**Table 2.** Baseline characteristics of subjects by biscuits group following intention-to-treat analysis

Baseline characteristics	Fortified biscuits (*n* 309)		Unfortified biscuits (*n* 312)	
	Mean	SD	Mean	SD
Vital and personal characteristics				
Age, years^*^.	12·8	1·9	12·8	2·0
Height-for-age *Z*-score (HAZ)^*^	−0·9	1·2	−1·0	1·1
	*n*	%	*n*	%
Stunted (HAZ < −2 SD), %	51	16·5	57	18·3
	Mean	SD	Mean	SD
BMI-for-age *Z*-score (BAZ)^*^	−0·8	0·9	−0·7	0·8
	*n*	%	*n*	%
BMI *Z*-score category, %				
Underweight (BAZ < −2 sd)	24	7·8	20	6·4
Overweight/obese (BAZ > + 1 SD)	4	1·3	5	1·6
Positive for *Plasmodium* infection, %	126	40·8	125	40·1
	Median	25^th^ percentile, 75^th^ percentile	Median	25^th^ percentile, 75^th^ percentile
Inflammation markers				
C-reactive protein (CRP), mg/l^†^	0·2	0·1, 0·7	0·2	0·1, 0·8
*α*-1-acid glycoprotein (AGP), g/l^†^	0·9	0·6, 1·4	0·9	0·6, 1·4
	*n*	%	*n*	%
Inflammation, %				
CRP > 5 mg/l	17	5·5	22	7·1
AGP > 1 g/l	131	42·4	132	42·3
Inflammation (CRP > 5 mg/l or AGP > 1 g/l)	133	43·0	134	43·0
Inflammation category, %				
Reference	176	57·0	178	57·1
Incubation	2	0·7	2	0·6
Early convalescence	15	4·9	20	6·4
Late convalescence	116	37·5	112	35·9
	Mean	SD	Mean	SD
Haemoglobin status and anaemia				
Haemoglobin, g/l^*^	120·0	10·2	120·0	10·2
	*n*	%	*n*	%
Anaemia^‡^ (haemoglobin < 115/120 g/l), %	121	39·2	132	42·3
Anaemia severity, %				
Mild^§^	73	23·6	73	23·4
Moderate (80 g/l ≤ haemoglobin ≤109 g/l)	48	15·5	59	18·9
	Median	25^th^ percentile, 75^th^ percentile	Median	25^th^ percentile, 75^th^ percentile
Micronutrient biomarkers and deficiencies after BRINDA adjustment				
Ferritin (PF), µg/l^†^	47·2	24·1, 68·0	44·7	26·9, 68·1
Transferrin receptor concentration (TfR), mg/l^†^	8·1	6·0, 11·6	8·4	6·0, 11·3
Retinol-binding protein (RBP), µmol/l^†^	1·2	0·9, 1·7	1·3	1·0, 1·8
	Mean	SD	Mean	SD
Body iron stores, mg/kg^*^	−20·7	7·8	−20·4	7·2
	*n*	%	*n*	%
Iron deficiency (PF < 15 µg), %	38	12·3	29	9·3
Tissue iron deficiency (TfR > 8·3 mg/l), %	150	48·5	161	51·6
Iron deficiency (PF < 15 µg/l and/or TfR > 8·3), %	165	53·4	170	54·5
Iron deficiency anaemia (anaemia with PF < 15 µg and/or TfR > 8·3 mg/l), %	64	21·0	80	25·6
Vitamin A deficiency (RBP < 0·7 µmol/l), %	35	11·3	23	7·4
Low or marginal vitamin A status (0·7 ≤ RBP < 1·05 µmol/l), %	71	23·0	71	22·8
Micronutrient deficiencies excluding children with inflammation, %				
Iron deficiency (PF < 15 µg)	27	15·3	18	10·1
Tissue iron deficiency (TfR > 8·3 mg/l)	93	52·8	93	52·3
Iron deficiency (PF < 15 µg/l or TfR > 8·3)	107	60·8	102	57·3
Iron deficiency anaemia (anaemia with PF < 15 µg and/or TfR > 8·3 mg/l)	47	26·70	40	22·5
Vitamin A deficiency (RBP < 0·7 µmol/l)	31	17·6	24	13·5
Low or marginal vitamin A status (0·7 ≤ RBP < 1·05 µmol/l)	43	24·4	41	23·0
Iron deficiency with unadjusted PF, %				
PF < 15 µg/l for girls without SCI but PF < 70 µg/l for girls with SCI^‖^	79	25·6	74	23·7

HAZ, height-for-age *Z*-score; BAZ, BMI-for-age *Z*-score; AGP, *α*-1-acid glycoprotein; CRP, C-reactive protein; PF, plasma ferritin; TfR, Transferrin receptor concentration; RBP, retinol-binding protein; BRINDA, biomarkers reflecting inflammation and nutritional determinants of anaemia.

* Values are means ± sd.

† Values are the median (25^th^ percentile, 75^th^ percentile)

‡ Anaemia, haemoglobin status < 115 g/l for girls aged <12 years and haemoglobin < 120 g/l for girls aged ≥12 years.

§ Mild anaemia: 110 g/l ≤ haemoglobin ≤114 g/l for girls aged 10–11 years and 110 g/l≤ haemoglobin ≤119 g/l for girls aged ≥12 years.

‖ Definition of iron deficiency using the unadjusted plasma ferritin following the WHO guidelines^(39)^.

Where specified as %, values are frequencies with the percentage in brackets.

**Table 3.** Baseline maternal and household-related characteristics of the subjects by biscuits group following intention-to-treat analysis

Variable	Fortified biscuits group (*n* 309)		Unfortified biscuits group (*n* 312)	
	*n*	%	*n*	%
Maternal characteristics				
Mother is aged 50 years. and above (%)	279	90·3	285	91·4
Mother is literate (%)	31	10·0	17	5·5
	Mean	sd	Mean	sd
Final decision-making index of mother	5·3	1·3	5·4	1·3
	*n*	%	*n*	%
Household characteristics				
Household food security (%)				
Food-secure	64	20·7	49	15·7
Food insecure	245	79·3	263	84·3
Household wealth index (%)				
Quintile 1	56	18·1	68	21·8
Quintile 2	76	24·6	48	15·4
Quintile 3	51	16·5	61	19·6
Quintile 4	67	21·7	54	17·3
Quintile 5	59	19·1	54	17·3

Values are frequencies and percentages in the bracket except where specified.

### Intervention effect on biomarkers of micronutrient status

After 26 weeks of intervention, we found no difference in PF, TfR and RBP in the MMB group compared with the UB group (Table 4). Post-intervention Hb (-1·2; 95 % CI (3·0, 0·6) g/l) decreased marginally in the MMB compared with the UB group, unexpectedly (Table 4). When we stratified by menarche status, we found that pre-menarche girls had a modest gain in Hb status (0·4; 95 % CI (−2·1, 3·0) g/l), whereas post-menarche girls had a decline (-2·8, 95 % CI (−5·4, −0·3) g/l). In either pre-menarche or post-menarche girls, no apparent variations in PF, TfR, or RBP were identified between the groups. We obtained similar results when we repeated the analysis using the per-protocol criteria (online Supplementary Table S2) and in the subgroup analysis for girls who were anaemic at baseline (online Supplementary Table S3). A stratified analysis by baseline deficient/low vitamin A status revealed that vitamin A and menarche status influenced the intervention’s effect, with MMB post-menarche girls having a substantial increase in post-intervention RBP (12·6 %, 95 % CI (0·3, 25·0)) compared with their peers in the UB (online Supplementary Table S4).

**Table 4.** The effect of consuming micronutrient-fortified biscuits compared with unfortified biscuits on micronutrient biomarkers after 26 weeks of intervention in adolescent girls in Ghana: an intention-to-treat analysis

Outcome	Baseline						After 26 weeks						The post-intervention difference in Means (MMB-UB)					
	Overall (*n* 621)		Pre-menarche (*n* 312)		Post-menarche (*n* 309)		Overall		Pre-menarche		Post-menarche		Overall sample (*n* 621)		Pre-menarche (*n* 312)		Post-menarche (*n* 309)	
	Mean	SD	Mean	SD	Mean	SD	Mean	SD	Mean	SD	Mean	SD	Prevalence difference	95 % CI	Prevalence difference	95 % CI	Prevalence difference	95 % CI
Ferritin (µg/l)^*^																		
UB	41·26	2·05	45·60	1·97	37·0	2·1	36·6	2·5	40·9	1·9	32·8	2·1	Ref.		Ref.		Ref.	
MMB	39·65	2·16	43·82	2·03	36·2	2·3	35·5	2·1	39·3	1·9	32·1	2·3	0·3	−7·2, 7·8	0·4	−10·2, 11·0	0·2	−10·4, 10·8
Soluble transferrin receptor (mg/l)^*^																		
UB	8·2	1·7	8·33	1·68	8·0	1·6	9·0	1·6	9·1	1·6	8·9	1·6	Ref.		Ref.		Ref.	
MMB	8·1	1·7	8·41	1·57	7·8	1·8	9·2	1·6	9·2	1·5	9·3	1·7	2·6	−3·9, 9·0	−0·1	−9·3, 9·0	5·3	−3·9, 14·5
Retinol-binding protein (µmol/l)^*^																		
UB	1·2	1·7	1·26	1·68	1·4	1·6	1·2	1·4	1·2	1·4	1·2	1·4	Ref.		Ref.		Ref.	
MMB	1·3	1·4	1·22	1·60	1·3	1·7	1·2	1·4	1·1	1·4	1·2	1·4	0·6	−4·3, 5·5	−2·6	−9·6, 4·3	3·8	−3·2, 10·8
Body iron store (mg/kg)																		
UB	−20·4	7·2	−19·73	7·13	−21·2	7·3	−22·3	7·5	−21·5	7·10	−23·1	7·9	Ref.		Ref.		Ref.	
MMB	−20·7	7·8	−20·20	6·99	−21·1	8·5	−22·7	8·0	−21·8	6·7	−23·6	9·0	−0·2	−0·9, 0·5	0·1	−0·9, 1·1	−0·5	−1·5, 0·6
Haemoglobin concentration (g/l)																		
UB	119·6	12·0	118·5	11·9	120·8	12·0	120·9	12·8	119·6	12·9	122·2	12·6	Ref.		Ref.		Ref.	
MMB	120·4	12·2	119·9	11·9	120·9	12·4	120·0	12·7	120·6	11·4	119·4	13·9	−1·2	−3·0, 0·6	0·4	−2·1, 3·0	−2·8	−5·4, −3·0^*^

MMB, multiple micronutrient-fortified biscuits; UB, unfortified biscuits.

* Outcomes variables were log-transformed (Ln), means are geometric means, and estimates were expressed as percentages increase or decrease.

*P-value* < 0·05.

Since some of the pre-menarche girls attained menarche before the end of the intervention, we repeated the analysis after re-classifying the girls at the endline as pre-to-pre-menarche, pre-to-post-menarche and post-to-post-menarche but similar results were observed for the pre-to-pre- and post-to-post-menarche groups. Although we observed an improvement in PF for MMB *v*. UB (1·3 %, 95 % CI (1·0, 1·5)) for the pre-to-post-menarche subgroup for subjects who were anaemic at baseline, the analysis was limited by the smaller sample size (*n* 14) for the subgroup.

### Intervention effect on micronutrient deficiencies

Post-intervention anaemia decreased slightly by 2·3 % among pre-menarche girls in the MMB group compared with UB. However, post-menarche girls in the MMB group experienced a marginal increase of 9·7 % in anaemia prevalence compared with UB (Table 5). The analysis revealed significant interaction effects between biscuits and menarche for post-intervention VAD (*P* = 0·04) and deficient/low vitamin A status (*P* = 0·03). After adjusting for baseline age, HAZ and micronutrient biomarkers (Hb PF, TfR and RBP), the prevalence of VAD increased by 6·2 % points among pre-menarche girls in the MMB group compared with UB. On the other hand, MMB had a slightly positive effect on post-menarche girls, showing a 9·6 % points lower prevalence of deficient/low vitamin A status compared with UB. Similar results were observed in the per-protocol analysis (online Supplementary Table S5). In the subgroup analysis for participants with anaemia at baseline (*n* 253), the prevalence of anaemia was 14·9 % higher in post-menarche girls on MMB compared with UB, and the prevalence of IDA was 13·4 % points higher (online Supplementary Table S6).

**Table 5.** The effect of consuming micronutrient-fortified biscuits compared with unfortified biscuits on post-intervention prevalence difference in micronutrient deficiencies after 26 weeks of intervention in adolescent girls in Ghana: an intention-to-treat analysis

Outcome	Prevalence rate of micronutrient deficiency for the overall population				Post-intervention in micronutrient deficiency (MMB-UB)					
	Baseline		Post-intervention		Overall sample (*n* 621)		Pre-menarche (*n* 312)		Post-menarche (*n* 309)	
	MMB *n* 309	UB *n* 312	MMB *n* 309	UB *n* 312	Prevalence difference^*^	95 % CI	Prevalence difference^*^	95 % CI	Prevalence difference^*^	95 % CI
Anaemia	39·2	42·3	42·1	39·1						
Model 1^†^					3·0	-4·7, 10·7	-2·7	−13·5, 0·1	8·7	−2·3, 19·7
Model 2^‡^					4·1	−3·1, 11·2	−2·3	−12·6, 8·0	9·7	−0·2, 19·6
Iron deficiency (PF < 15 µg/l)	12·3	9·3	12·0	10·3						
Model 1					1·7	−3·2, 6·6	0·74	−5·1, 6·5	2·7	−5·3, 10·6
Model 2					1·3	−2·7, 5·3	2·39	−2·4, 7·2	1·1	−5·0, 7·2
Tissue iron deficiency (TfR > 8·3 mg/l)	48·5	51·6	53·7	51·0						
Model 1					2·8	−5·1, 10·6	5·2	−5·9, 16·2	0·3	−10·8, 11·5
Model 2					2·3	−4·9, 9·5	3·5	−6·7, 13·8	1·7	−8·38, 11·8
Iron deficiency (PF < 15 µg/l or TfR > 8·3 mg/l)	53·4	54·5	55·7	54·2						
Model 1					1·5	−6·3, 9·3	2·62	−8·4, 13·7	0·4	−10·7, 11·4
Model 2					1·1	−5·9, 8·2	1·06	−9·1, 11·2	1·8	−7·94, 11·6
IDA (anaemia and PF < 15 µg/l or TfR > 8·3 mg/l)	21·0	25·6	24·9	20·8						
Model 1					4·1	−2·5, 10·7	0·9	−8·3, 10·1	7·3	−2·2, 16·7
Model 2					4·5	−1·3, 10·3	1·8	−6·8, 10·3	7·4	−0·3, 15·2
VAD (RBP < 0·7µmol/l)	11·3	7·4	6·2	3·9						
Model 1					2·3	−1·1, 5·7	5·86	0·1, 11·6	−1·28	−4·8, 2·3
Model 2					2·0	−1·3, 5·3	6·15	0·7, 11·6	−1·31	−4·9, 2·3
Low or VAD (RBP < 1·05 µmol/l)	34·3	30·1	35·9	37·5						
Model 1					−1·6	−9·1, 6·0	4·4	−6·5, 15·3	−7·6	−18·0, 2·8
Model 2					−2·4	−9·3, 4·6	5·2	−5·1, 15·6	−9·6	−18·9, −0·3

MMB, multiple micronutrient-fortified biscuits; UB, unfortified biscuits.

* All results are in percentages, reflecting the percentage point difference between the fortified compared with the unfortified biscuits group.

† Model 1 included the biscuits group and the study design effect (menarche status at enrolment).

‡ Model 2 adjusted for baseline micronutrient biomarkers (haemoglobin, PF, TfR and RBP) and the girl’s baseline age and HAZ.

## Discussion

In this study, we hypothesised that MMB compared with UB consumption 5 d weekly for 26 weeks would improve adolescent girls’ micronutrient status. We also explored to what extent the intervention effect varied before or after menarche. Contrary to our hypothesis, we found no evidence that MMB consumption improves iron and haemoglobin status. Our study population had a low socio-economic status with high household food insecurity, and the prevalence of anaemia was higher than that reported by Gosdin *et al.*^(8)^ for adolescent girls aged 10–19 years in Ghana. Interestingly, we found that the effect of the intervention on vitamin A status and haemoglobin levels varied depending on participants’ menarche status and baseline vitamin A status. Post-menarche girls experienced a significant drop in deficient/low vitamin A status, around 10 % points, although this improvement did not translate to a significant improvement in haemoglobin levels or anaemia.

A modest improvement in ferritin meant that post-intervention IDA did not decrease in the MMB group compared with the UB group. In conformity with Righetti *et al.*^(45)^, the prevalence estimates of ID and IDA were particularly higher when using or including TfR. Consequently, caution may be needed when estimating ID and IDA prevalence in a context with probable high SCI like Ghana.

Our findings contrast with the results of the meta-analysis by Das *et al*.^(46)^, which showed significant improvements in haematologic biomarkers and micronutrient concentrations with food fortification. Instead, our study supports the more recent systematic review and meta-analyses conducted by Eichler *et al.*^(47)^ and Salam et al.^(48)^, indicating that the consumption of fortified dairy and cereal foods may only lead to minor increases in Hb levels without significant differences in anaemia risk among children. Although the duration of our investigation was only 26 weeks (6 months), several other studies^(21,25)^ found that MMF had a substantial impact on the micronutrient status of children and adolescents when eaten for an average of 5 d/week for 6 months.

Our findings highlight the complex interplay of various factors, such as iron compound choice and dose, dietary patterns, infection and inflammation, in determining the efficacy of micronutrient interventions. In the present study, ferrous fumarate was the fortificant used for iron, while dry vitamin A palmitate (retinyl palmitate) was used for vitamin A, both of which have been suggested by the WHO/FAO for the fortification of wheat flour^(17)^. Zimmermann *et al.*^(49)^ hypothesised that an additional intake of 23 mg of iron/d is necessary to significantly improve the iron status of children who consume predominantly cereal-legume diets, like our study population. Given that the MMB provided only 4·1mg of iron per serving, it is likely that this amount was insufficient to improve the iron status of our study population effectively. The MMB used in our study was already available on the market and was designed to meet only 15 % and 30 % of the recommended dietary allowance of iron and vitamin A, respectively, for women aged 19–35 years. Our study results indicate that such a product is not capable of enhancing the micronutrient status of adolescent girls within a 6-month intervention period. An additional factor that might have limited the impact of the MMB was the mandatory fortification of wheat flour in Ghana; it led to a small difference of only 3·1 mg of iron per serving between the MMB and the UB, reducing the power of our study to detect significant changes in iron status.

No difference was observed in the TfR of girls receiving MMB compared with girls receiving UB after the intervention, indicating poor iron erythropoiesis^(50)^. This shows that tissue ID is ongoing, which could be due to compensatory reductions in circulating iron (PF) and helps to explain why the MMB group’s Hb did not improve in our study. Aside from the loss for post-menarche girls, our study’s observed decline in Hb suggests that bone marrow erythropoiesis could not keep up with the rate of blood volume expansion^(50)^. Furthermore, iron losses associated with blood loss and the pro-inflammatory nature of menstruation^(9)^ are likely to have a worse effect on iron absorption than any gains from compensatory absorption during menstrual loss, which is a potential explanation for the decline in Hb observed in post-menarche girls. Furthermore, we expected to see a more significant intervention effect of the MMB in the subgroup of girls who had anaemia or ID at baseline because iron absorption is typically up-regulated deficiency^(13)^ when there is an existing deficiency. However, this expectation was not met, suggesting inadequate iron erythropoiesis even in a deficient state. The absence of an effect on PF and TfR in the present study could explain the slight drop in body iron stores in the MMB group compared with the UB group.

The lack of effect on RBP and vitamin A status indicators in the study cannot be solely explained by the dosage used as comparable studies^(25,51)^, used a similar dose; other intrinsic and extraneous factors like underlying infections and inflammation may be influencing both vitamin A and iron status indicators. Infections affect vitamin A and iron metabolism, increasing PF and TfR concentrations and decreasing plasma retinol concentrations, giving false-negative results^(31,52)^. A comparable efficacy trial with a greater dose (20 mg) of iron-fortified biscuits (4 d/week for 6 months) found no effect on haematologic biomarkers among 6–14 years old Ivorian school children (44 % girls), which the authors attributed in part to a higher occurrence of infections^(53)^; the baseline prevalence of SCI in our study was higher (43 % *v*. 29 %) than reported in this study. Despite deworming all participants at the start, our sample’s post-intervention SCI prevalence remained high, and it was mostly in the late convalescence stage, indicating chronic infection and inflammation. In a national survey, SCI and VAD each accounted for about 10 % of anaemia among reproductive-age women in Ghana^(54)^. Considering the above, efficacy trials that include iron and vitamin A may need to exclude subjects with SCI. Addressing inflammation and strengthening deworming efforts could be essential in improving the efficacy of interventions targeting vitamin A and iron status among school children.

Asymptomatic malaria can have a significant impact on haematologic and micronutrient biomarkers; it reduces Hb and RBP levels while increasing PF and TfR concentrations^(55)^. In the present study, the observed increase in TfR levels post-intervention may also be a response to haemolysis caused by malaria^(56)^. The endline assessment occurred during the peak of the rainy season, which was associated with a minor increase in malaria prevalence (from 40 % to ∼53·5 %). Our mid-point validation of the malaria rapid test kit in a sub-sample (*n* 68) of the girls showed poor sensitivity (sensitivity = 13·3 %; specificity = 84·9 %; *results not shown*). Consequently, the adjustment for malaria in the analysis of micronutrient biomarkers might have been incomplete, leading to a potential measurement error. Although the randomised design of the study helped prevent systemic bias, the incomplete adjustment for malaria could have influenced the study’s power to some extent. Efficacy trials in malaria-endemic contexts should consider malaria microscopy for assessing malaria status when logistically possible.

Apart from ID, other micronutrient deficiencies like folate, riboflavin, vitamin A, B_6_, B_12_, zinc and copper are potential contributors to anaemia^(57,58)^ but the MMB used in the study included these nutrients. Genetic factors such as haemoglobinopathies may also partly account for the modest effects on iron and anaemia. Although the prevalence of sickle cell traits among Ghanaian adolescents is unknown, about a third of infants and young children, and non-pregnant reproductive-age women in Ghana have *α*-thalassaemia, with more than a tenth having sickle cell disorder^(7)^. Sickle cell traits may be associated with increased TfR^(59)^. But as we did not assess Hb variants, we are unable to examine the extent to which these conditions contributed to elevated TfR in our population.

The decline in Hb in our post-menarche girls and the improvement in vitamin A status suggest that, in some circumstances, vitamin A improvement may not affect Hb. For instance, Leenstra *et al.*^(60)^ in Western Kenya demonstrated that weekly vitamin A supplementation had no effect on adolescent schoolgirls’ ferritin levels (-1·7 µg/l, 95 % CI (−5·4, 2·7)) or haemoglobin levels (-0·7 g/l, 95 % CI (−3·8, 2·5)). On the other hand, the increase in VAD after MMB consumption in pre-menarche girls suggests that VAD might not be the main factor causing VAD in this population in our setting. In children and adolescents, infection resistance improves with age^(61)^, therefore, pre-menarche females are likely to be more vulnerable to the effects of infections on their vitamin A status. Although we found no differences in SCI and *Plasmodium* infection by menarche status at baseline and endline, pre-menarche girls were more likely to have *Plasmodium* infection during the intervention. Overall, our results imply that menarche status affects vitamin A status but further research specific to menarche status and micronutrient absorption is needed to inform public health recommendations.

The difference in Hb measurement between capillary (finger prick at baseline) and venous blood (at endline) might have caused a systematic overestimation of baseline Hb levels in the study^(62)^. Importantly, this bias was consistent across both intervention and control groups; as a result, it is less likely to have influenced the conclusions drawn from the study regarding the impact of the MMB on Hb levels.

Retinol remains the recommended biomarker for assessing the vitamin A status of populations; RBP is an acute-phase protein, and its levels can be affected by factors such as protein-energy malnutrition, infection and inflammation^(63)^. It was therefore probable that RBP did not accurately reflect the true vitamin A status of the girls. Nevertheless, RBP has been demonstrated to yield an unbiased estimate of VAD when combined with CRP^(64)^.

While the study focused on rural adolescent girls, caution should be exercised when extrapolating the results to populations with different nutritional profiles or health conditions. In this group of rural Ghanaian adolescent girls, MMB consumption did not improve Hb and iron status, but it reduced the prevalence of deficient/marginal vitamin A status among post-menarche girls. Despite the modest effects observed in our study, food fortification programmes remain relevant considering the high burden of anaemia, ID and IDA in the present study. Micronutrient supplements may be a better approach, but poor compliance still limits their effectiveness^(65)^. Hence, longer-term consumption of fortified foods alongside regular treatment of infections may be critical for improving micronutrient status.

## Supplementary material

For supplementary material accompanying this paper visit https://doi.org/10.1017/S0007114523002234. click here to view supplementary material
