# Supplementary material for: Ten2Twenty-Ghana: a randomised controlled trial on the efficacy of multiple micronutrient-fortified biscuits on the micronutrient status of adolescent girls
Source: Br J Nutr. 2023 Oct 5;131(4):707–19. doi: 10.1017/S0007114523002234 (PMC10803820; doi:10.1017/S0007114523002234)
Supplement: Supplementary file 1 [file S0007114523002234sup001.docx]

**Supplementary Material for the Manuscript: Ten2Twenty-Ghana: A Randomized Controlled Trial on the Efficacy of Multiple-Micronutrient Fortified Biscuits on the Micronutrient Status of Adolescent Girls**

# SUPPLEMENTARY METHODS

**Table S1: The Inclusion and Exclusion Criteria for Selection into the Ten2Twenty-Ghana Study**

| **No** | **Inclusion criteria for the survey** | **Exclusion criteria for Randomised Controlled Trial** |
| --- | --- | --- |
| 1 | Aged 10 to 17 years (verified by birth certificate, health- record, insurance card, school register, or another formal document) | Severely anaemic (Hb < 80 g/L ^(1)^)^1^ |
| 2 | Apparently healthy without any visible sign(s) of poor health | History of medical/surgical events that may significantly affect RCT outcomes |
| 3 | Non-pregnant | Sign(s) of chronic infection or metabolic disorder |
| 4 | Non-lactating | Clinical sign(s) of vitamin A deficiency and/or iodine deficiency |
| 5 | No incompatible mental status | Severely underweight (BAZ^2^ < – 3SD) |
| 6 | Willing to participate | Taking medical drugs or nutrient supplements at the time of enrolment |
| 7 | Informed consent of parent or guardian obtained for the survey | Participating in another food-, supplement-, and/or drug study |
| 8 |  | Not willing to consume biscuits from Monday to Friday for 26 weeks |
| 9 |  | Any known food allergy to biscuits |
| 8 |  | Afraid or not willing to donate approximately 12 mL of blood on 2 different occasions |
| 9 |  | Refusal of parents or guardian |
| 10 |  | ^3^Second informed consent from parent or guardian obtained for RCT |

^1^Hb: Haemoglobin; those who were severely anaemic were referred to a hospital; ^2^BAZ: Body Mass Index- for-age z-scores; ^3^Ethical approval requirements demanded that we obtain 2 different informed consents for the extensive survey and the RCT

## Details of Covariates

24-hour Dietary Recall: A single qualitative 24-hour dietary recall (24hR) assessed the dietary diversity score (DSS) of the girls using a 10-food group indicator ^(2)^. In the 24hR, the girl was first asked to mention all foods, drinks, and snacks that she consumed in and outside the home (including school) the previous day. She was then asked to describe the ingredients of any mixed dishes. Based on a pre-defined table with a list of all possible food items in the 10 food groups, a score of 1, else 0 was given if a girl consumed at least one food item from any food group. A summated score was computed by summing the scores for all the food groups, resulting in a maximum attainable score of 10. The 10 food groups included: grains, white roots and tubers, and plantains (1), pulses (beans, peas, and lentils) (2), nuts and seeds (3), dairy (4), meat, poultry, and fish (5), eggs (6), dark green leafy vegetables (7), other vitamin A-rich fruits and vegetables (8), other vegetables (9) and other fruits (10). We next defined minimum dietary diversity (MDD-W) as DDS ≥5 ^(2)^. We explore the effect of dietary diversity as a continuous score (DDS) and as a dichotomous variable (MDD-W).

Food frequency: Our data included the frequency (range 0-30 days) of the consumption of (1) animal source foods (eggs, fish, meat, dairy products), (2) legumes/nuts/seeds, (3) vitamin A-rich dark green leafy vegetables, and (4) other vitamin A-rich fruits and vegetables.

Food Security: We assessed the food insecurity status of the household with the Food Insecurity Experience Scale (FIES) (^3)^. The FIES is a tool to measure the severity of food insecurity on a continuum and consists of 8 questions with response categories “yes” or “no”. A summated score ranging from 0-8 was created in which a “yes response” was scored 1 and a “no response” was scored 0. Based on the summated score, the households were classified as food secure (score=0), mild food insecure (score=1-3), moderate food insecure (score=4-6) and severe food insecure (score=7-8). We subsequently re-categoirsed subjects as food secure (score =0) or food insecure (score ≥ 1).

Household Wealth Index: We created a household asset index using principal component analysis (PCA) and then ranked subjects' households into quintiles of household wealth ^(4)^. The index was based on the ownership of durable assets including TV, refrigerator, phone, bicycle, car, and household utensils categorized as low-cost (<50 USD) and expensive (> 300 USD), access to electricity, the type of water and toilet facilities accessed by the household and the floor material of the household. The Kaiser- Meyer-Olkin test for sampling adequacy (KMO) for the PCA was 0.94, and each of the separate items had a KMO of at least 0.88; higher than the acceptable limit of 0.5 ^(5)^. The first-factor score, with an eigenvalue of 7.7 and accounting for 64% of the variance in the data, was used to rank the households into quintiles of the wealth index.

## Internal regression correction of micronutrient biomarkers for inflammation

We used the Internal Regression Correction (IRC) approach ^(6–8)^ of the BRINDA group to adjust PF, TfR and RBP for inflammation biomarkers (CRP and AGP) and *Plasmodium* infection. In the IRC approach, linear regression is used to adjust a biomarker by the concentration of C-reactive protein (CRP) and alpha-1-acid glycoprotein (AGP) on a continuous scale and *Plasmodium* infection as a dichotomous variable. Natural log transformation was applied to linearize the relationship between plasma nutrient markers and inflammatory markers. We excluded subjects with CRP below and/or TfR and RBP above the detection limit. We adjusted TfR for AGP and *Plasmodium* infection but not for CRP. The variance inflation factors were < 2, suggesting no multicollinearity between predictors in the regression models. The lowest CRP decile at baseline and end surveys was the same as the external reference value (0.10mg/L). However, our AGP reference values (baseline=0.36 g/L and end-line=0.40 g/L) were less than the reference values (0.54 g/L and 0.59 g/L) of the BRINDA group (6). Considering that the majority (81.2%) of our study population was below 15 years, we used the external reference values of CRP (0.10mg/L) and AGP (0.59g/L) for the preschool-aged children from the BRINDA group (6). The linear regression equation used in the present study to adjust the plasma indicators is shown below.

LnMB_adj_=LnMB_unadj_ - β_1_(Ln CRP_obs_-LnCRP_ref_) - β2(LnAGP_obs_-LnAGP_ref_) - β_3_**Plasmodium* infection.

Where MB, represents the micronutrient biomarker, the subscripts adj and unadj represent “*adjusted”* and “*unadjusted*” while the subscripts obs and ref represent “*observed*” and “*reference*”.

**SUPPLEMENTARY RESULTS**

**Table S2: The effect of MMB versus UB on micronutrient status after 26 weeks of intervention in adolescent girls in Ghana: A per-protocol analysis**

| **Outcome** | **Mean ± SD at Baseline** | | | **Mean ± SD after 26 weeks** | | | **The post-intervention difference in Means**  **(MMB-UB)** | | |
| --- | --- | --- | --- | --- | --- | --- | --- | --- | --- |
|  | **Overall (n=559)** | **Pre-menarche (n=282)** | **Post-menarche (n=277)** | **Overall (n=559)** | **Pre-menarche (n=282)** | **Post-menarche (n=277)** | **Overall (n=559)** | **Pre-menarche (n=282)** | **Post-menarche (n=277)** |
| **Ferritin (µg/L)^1^** |  |  |  |  |  |  |  |  |  |
| UB | 41.7 ± 2.1 | 45.6 ± 2.0 | 38.1 ±2.1 | 36.6 ± 2.0 | 40.9 ±1.9 | 32.8 ± 2.1 | Ref. | Ref. | Ref. |
| MMB | 39.7 ± 2.2 | 43.4 ± 2.1 | 36.3 ±2.3 | 35.2 ± 2.1 | 38.9 ±1.9 | 31.5 ±2.2 | -0.7% (-8.5%, 7.1%) | -0.9% (-11.9%, 10.1%) | -0.5% (-11.6%, 10.6%) |
| **Soluble transferrin receptor (mg/L)^1^** |  |  |  |  |  |  |  |  |  |
| UB | 8.3 ± 1.7 | 8.33 ± 1.7 | 8.2 ± 1.6 | 9.0 ± 1.6 | 9.2 ± 1.6 | 8.9 ± 1.6 | Ref. | Ref. | Ref. |
| MMB | 8.2 ± 1.7 | 8.50 ±1.6 | 7.9 ± 1.8 | 9.2 ± 1.6 | 9.2 ±1.5 | 9.2 ± 1.7 | 2.3% (-4.5%, 9.1%) | 0.2% (-9.4%, 9.7%) | 4.42% (-5.2%, 14.1%) |
| **Retinol binding protein (µmol/L)^1^** |  |  |  |  |  |  |  |  |  |
| UB | 1.3 ± 1.7 | 1.3 ± 1.7 | 1.4 ± 1.6 | 1.2 ± 1.4 | 1.2 ± 1.4 | 1.2 ± 1.4 | Ref. | Ref. | Ref. |
| MMB | 1.3 ±1.7 | 1.2 ± 1.6 | 1.3 ±1.8 | 1.2 ± 1.4 | 1.1± 1.4 | 1.2 ±1.4 | -0.4% (-5.5%, 4.7%) | -4.5% (-11.7%, 2.6%) | 3.8% (-3.4%, 11.0%) |
| **Body iron store (mg/kg)** |  |  |  |  |  |  |  |  |  |
| UB | -20.5± 7.3 | -19.8 ±7.2 | -21.2±7.3 | -22.3 ± 7.6 | -21.5±7.3 | -23.1± 8.0 | Ref. | Ref. | Ref. |
| MMB | -20.8 ±7.9 | -20.3 ±7.1 | -21.2 ±8.7 | -22.8 ± 8.0 | -21.9 ±6.8 | -23.6 ±9.1 | -0.2 (-1.1, 0.5) | -0.03 (-1.1, 1.1) | -0.5 ( -1.6, 0.6) |
| **Haemoglobin concentration (g/L)** |  |  |  |  |  |  |  |  |  |
| UB | 119.5 ± 12.0 | 118.5 ±12.0 | 120.6 ±11.9 | 120.7 ± 12.6 | 119.9 ±12.9 | 121.4±12.2 | Ref. | Ref. | Ref. |
| MMB | 120.2 ±12.5 | 119.2 ±12.1 | 121.2 ±12.7 | 120.2 ± 12.7 | 120.6 ±11.3 | 119.7 ±13.9 | -0.8 (-2.7, 1.1) | 0.4 (-2.3, 3.0) | -2.0 (-4.7, 0.7) |

^1^Outcomes variables were log-transformed (Ln), means are geometric means and estimates were expressed as a percentage increase or decrease. *MMB, multiple-micronutrient fortified biscuits; UB, unfortified biscuits*

**Table S3: The effect of MMB versus UB on micronutrient status after 26 weeks of intervention in anaemic adolescent girls in Ghana: An intention-to-treat analysis**

| **Outcome** | **Mean ± SD at Baseline** | | | **Mean ± SD after 26 weeks** | | | **The post-intervention difference in Means**  **(MMB-UB)** | | |
| --- | --- | --- | --- | --- | --- | --- | --- | --- | --- |
|  | **Overall sample (n=253)** | **Pre-menarche (n=132)** | **Post-menarche (n=121)** | **Overall sample (n=253)** | **Pre-menarche (n=132)** | **Post-menarche (n=121)** | **Overall sample (n=253)** | **Pre-menarche (n=132)** | **Post-menarche (n=121)** |
| **Ferritin (µg/L)^1^** |  |  |  |  |  |  |  |  |  |
| UB | 36.2 ± 2.2 | 39.3 ± 2.2 | 33.8 ± 2.1 | 31.8 ± 2.2 | 34.1 ± 2.1 | 29.7 ± 2.3 | Ref. | Ref. | Ref. |
| MMB | 37.3 ± 2.2 | 42.5 ± 2.1 | 32.8 ± 2.3 | 33.5 ± 2.3 | 38.9 ± 2.0 | 29.4 ± 2.4 | 5.4% ( -6.2%, 17.6%) | 9.5% (-8.3%, 2.7%) | 1.3% (-15.5%, 18.1%) |
| **Soluble transferrin receptor (mg/L)^1^** |  |  |  |  |  |  |  |  |  |
| UB | 9.0 ± 1.7 | 9.3 ± 1.7 | 8.8 ± 1.6 | 9.7 ± 1.7 | 10.1 ± 1.7 | 9.3 ± 1.6 | Ref. | Ref. | Ref. |
| MMB | 8.5 ± 1.8 | 8.9 ± 1.6 | 8.2 ±1.9 | 9.9 ± 1.7 | 9.9 ± 1.5 | 10.0 ±1.8 | 4.9% (-6.0%, 15.8%) | -0.30% (-16.0%, 15.5%) | 10.2% (-4.8%, 25.1%) |
| **Retinol binding protein (µmol/L)^1^** |  |  |  |  |  |  |  |  |  |
| UB | 1.28 ± 1.6 | 1.2 ± 1.6 | 1.4 ± 1.5 | 1.1 ± 1.4 | 1.1 ± 1.4 | 1.2 ± 1.3 | Ref. | Ref. | Ref. |
| MMB | 1.2 ± 1.7 | 1.2 ± 1.7 | 1.3 ± 1.7 | 1.1 ± 1.4 | 1.1 ± 1.3 | 1.2 ± 1.4 | 1.4% (-5.8%, 8.5%) | -2.2% (-12.6%, 8.2%) | 4.9% (-5.0%, 14.8%) |
| **Body iron store (mg/kg)** |  |  |  |  |  |  |  |  |  |
| UB | -22.3 ± 8.0 | -21.9± 8.3 | -22.6 ± 7.7 | -24.0 ± 8.4 | -23.7 ± 7.9 | -24.2 ± 8.8 | Ref. | Ref. | Ref. |
| MMB | -21.6 ± 8.7 | -20.8 ± 7.1 | -22.3 ± 10.0 | -23.7 ± 8.9 | -22.42 ± 7.2 | -25.0 ± 10.1 | -0.3 (-1.5, -0.9) | 0.4 (-1.3, 2.1) | -1.01 (-2.76, 0.6) |
| **Haemoglobin concentration (g/L)** |  |  |  |  |  |  |  |  |  |
| UB | 108.8 ± 7 .7 | 107.1 ± 8.0 | 110.3 ± 7.1 | 117.5 ± 12.4 | 115.7 ± 12.0 | 119.0 ± 12.6 | Ref. | Ref. | Ref. |
| MMB | 109.0 ± 8.6 | 108.4 ± 8.3 | 109.5 ± 8.9 | 115.2 ± 13.4 | 116.4 ± 11.2 | 114.0 ± 15.0 | -2.4 (-5.3, 0.6) | -0.2 (-4.5,4.1) | -4.6 (-8.6, -0.5) * |

^1^Outcomes variables were log-transformed (Ln), means are geometric means and estimates were expressed as a percentage increase or decrease. *MMB, multiple-micronutrient fortified biscuits; UB, unfortified biscuits;* *^*^P-value < 0.05*

**Table S4: Effect of the MMB compared to UB on micronutrient status stratified by baseline vitamin A status: An intention-to-treat analysis**

|  | **Deficient or low vitamin A status at baseline**  **(RBP <1.05µmol/L)** | | | **Vitamin A sufficient at baseline**  **(RBP ≥1.05µmol/L)** | | |
| --- | --- | --- | --- | --- | --- | --- |
|  | **Overall (n=200)** | **Pre-menarche**  **(*n*=100)** | **Post-menarche (*n*=100)** | **Overall**  **(*n*=421)** | **Pre-menarche**  **(*n*=212)** | **Post-menarche**  **(n=209)** |
| **Ferritin (µg/L)^1^** |  |  |  |  |  |  |
| UB | Ref. | Ref. | Ref. | Ref. | Ref. | Ref. |
| MMB | -2.4% (-17.5%, 12.8%) | 2.6% (-18.6%, 23.7%) | -7.3% (-28.6%, 13.9%) | -0.9% (-9.3%, 7.5%) | -1.5% (-13.3%, 10.3%) | -3.8% (-12.3%, 11.5%) |
| **Soluble transferrin receptor (mg/L)^1^** |  |  |  |  |  |  |
| UB | Ref. | Ref. | Ref. | Ref. | Ref. | Ref. |
| MMB | 9.43% (-1.9%, 20.7%) | 9.73% (-6.1%, 25.6%) | 9.1% (6.7%, 24.9%) | -1.9% (-9.7%, 5.9%) | -4.8% (-15.7%, 6.2%) | 1.0% (-10.0%, 12.1%) |
| **Retinol binding protein (µmol/L)^1^** |  |  |  |  |  |  |
| UB | Ref. | Ref. | Ref. | Ref. | Ref. | Ref. |
| MMB | 5.8% ( -3.0%, 4.6%) | -1.1% ( -13.4%, 11.2%) | 12.6% (0.3%, 25.0%) * | -2.2% (-8,3%, 3.9%) | -3.3% (-11.8%, 5.3%) | -1.21% (-9.9%, 7.4%) |
| **Body iron store (mg/kg)** |  |  |  |  |  |  |
| UB | Ref. | Ref. | Ref. | Ref. | Ref. | Ref. |
| MMB | -0.1 (-0.2, 0.1) | -0.1 (-0.1, 0.1) | -0.1 (-0.3, 0.1) | -0.2 (-0.3, 0.0) * | -0.2 (-0.4, 0.0) | -0.1 (-0.4, 0.1) |
| **Hemoglobin status (g/L)^2^** |  |  |  |  |  |  |
| UB | Ref. | Ref. | Ref. | Ref. | Ref. | Ref. |
| MMB | -3.4 (-6.4, -0.3) * | 1.3 (-3.1, 5.6) | -8.0 (-10.23, -3.7) * | -0.03 (-2.15, 2.1) | 0.7 (-2.3, 3.7) | -0.8 (-3.77, 2.3) |

*MMB, multiple-micronutrient fortified biscuits; UB, unfortified biscuits;* **P-value <* 0.05; All biomarkers were adjusted for corresponding baseline values and the study design. ^1^Outcomes variables were log-transformed (Ln), and estimates were expressed as a percentage increase or decrease; ^2^Hemoglobin was additionally adjusted for baseline ferritin, transferrin receptor concentration, retinol-binding protein, and sub-clinical inflammation.

**Table S5: The effect of consuming micronutrient-fortified biscuits compared to unfortified biscuits on post-intervention prevalence difference in micronutrient deficiencies after 26 weeks of intervention in adolescent girls in Ghana: A per-protocol analysis**

| **Outcome** | **Prevalence rate of micronutrient deficiency for the overall population** | | | | **Post-intervention prevalence difference^1^ (95% CI) in micronutrient deficiency**  **(MMB-UB)** | | |
| --- | --- | --- | --- | --- | --- | --- | --- |
|  | **Baseline** | | **Post-intervention** | | **Overall sample (n=559)** | **Pre-menarche (n=282)** | **Post-menarche (n=277)** |
|  | **MMB (n=277)** | **UB**  **(n=282)** | **MMB (n=277)** | **UB**  **(n=282)** |  |  |  |
| **Anaemia** | 40.4 | 41.8 | 41.2 | 39.0 |  |  |  |
| Model 1 |  |  |  |  | 2.11 (-6.0, 10.2) | -2.2 (-13.5, 9.1) | 6.52 (-5.2, 18.2) |
| Model 2 |  |  |  |  | 3.2 (-4.4, 10.8) | -2.6 (-13.3, 8.2) | 7.87 (-2.7, 18.5) |
| **Iron deficiency (PF < 15µg/L)** | 13.4 | 8.9 | 12.3 | 9.9 |  |  |  |
| Model 1 |  |  |  |  | 2.3 (-2.9, 7.4) | 1.7 (-4.3, 7.6) | 2.9 (-5.6, 11.4) |
| Model 2 |  |  |  |  | 1.1 (-3.1, 5.2) | 2.5 (-2.4, 7.4) | 0.02 (-6.3, 6.4) |
| **Iron deficiency (TfR >8.3)** | 49.5 | 53.6 | 52.4 | 50.0 |  |  |  |
| Model 1 |  |  |  |  | 2.4 (-5.9, 10.7) | 5.4 (-6.3, 17.0) | -0.7 (-12.5, 11.1) |
| Model 2 |  |  |  |  | 2.2 ( -5.4, 9.7) | 3.4 (-7.4, 14.2) | 1.5 (-9.2, 12.1) |
| **Iron deficiency (PF <15µg/L or TfR >8.3)** | 54.5 | 56.4 | 54.5 | 53.2 |  |  |  |
| Model 1 |  |  |  |  | 1.3 (-7.0, 9.6) | 3.3 (-8.3, 14.9) | -0.7 (-12.5, 11.0) |
| Model 2 |  |  |  |  | 1.1 (-6.3, 8.6) | 1.4 (-9.3 12.1) | 1.71(-8.7, 12.7) |
| **IDA (anemia with PF <15µg/L or TfR >8.3)** | 21.7 | 26.6 | 24.2 | 20.2 |  |  |  |
| Model 1 |  |  |  |  | 4.0 ( -2.9, 10.9) | 2.9 (-6.7, 12.5) | 5.1 (-4.8, 15.0) |
| Model 2 |  |  |  |  | 4.2 (-1.8, 10.2) | 2.8 (-6.1, 11.6) | 5.3 (-2.8, 13.3) |
| **VAD (RBP < 0.7µmol/L)** | 11.6 | 7.5 | 6.1 | 4.8 |  |  |  |
| Model 1 |  |  |  |  | 1.9 (-1.7, 5.6) | 5.9 (-0.3, 12.2) | -2.2 (-5.9, 1.5) |
| Model 2 |  |  |  |  | 1.9 (-1.6, 5.5) | 6.6 (0.7, 12.5) | -2.1 (-5.8, 1.6) |
| **Low or VAD (RBP < 1.05 µmol/L)** | 32.5 | 29.4 | 37.2 | 37.6 |  |  |  |
| Model 1 |  |  |  |  | -0.3 (-8.3, 7.7) | 5.7 (-5.8, 17.29) | -6.5 (-17.69, 4.5) |
| Model 2 |  |  |  |  | -0.5 (-7.9, 6.8) | 6.6 (-4.2, 17.5) | -7.8 (-17.7, 2.1) |

^1^All results are in percentages, reflecting the percentage point difference between the fortified compared to the unfortified biscuits group. Model 1 included the biscuits group and the study design effect (menarche status at enrolment). Model 2 adjusted for baseline micronutrient biomarkers (haemoglobin, PF, TfR and RBP) and the girl's baseline age, and HAZ

**Table S6: The effect of consuming micronutrient-fortified biscuits compared to unfortified biscuits on post-intervention prevalence difference in micronutrient deficiencies after 26 weeks of intervention in adolescent girls in Ghana: A sub-group analysis among anaemic girls at baseline, following intention-to-treat**

| **Outcome** | **Prevalence rate of micronutrient deficiency for the overall population** | | | | **Post-intervention prevalence difference^1^ (95% CI) in micronutrient deficiency**  **(MMB-UB)** | | |
| --- | --- | --- | --- | --- | --- | --- | --- |
|  | **Baseline** | | **Post-intervention** | |  |  |  |
|  | **MMB (n=121)** | **UB (n=132)** | **MMB (n=121)** | **UB (n=132)** | **Overall sample (n=253)** | **Pre-menarche (n=120)** | **Post-menarche (n=133)** |
| **Anaemia** | 100 | 100 | 57.9 | 51.5 |  |  |  |
| Model 1 |  |  |  |  | 6.4 (-5.9, 18.6) | 0.02 (-17.6, 18.1) | 11.9 (-4.9, 28.7) |
| Model 2 |  |  |  |  | 8.5(-3.1, 20.0) | 1.8 (-15.7, 19.3) | 14.9 (0.2, 29.5) * |
| **Iron deficiency (PF < 15µg/L)** | 14.1 | 14.4 | 15.7 | 15.2 |  |  |  |
| Model 1 |  |  |  |  | 0.6 (-8.3, 9.5) | -0.8 (-12.7, 11.0) | 1.9 (-11.2, 15.0) |
| Model 2 |  |  |  |  | 11.7 (-5.9, 8.3) | 3.1 (-7.0, 13.1) | 0.34 (-9.2, 9.9) |
| **Iron deficiency (TfR >8.3 mg/L)** | 49.6 | 58.3 | 57.9 | 54.6 |  |  |  |
| Model 1 |  |  |  |  | 3.3 (-8.9, 15.5) | 3.8 (-13.9, 21.5) | 2.9 (-14.0, 19.8) |
| Model 2 |  |  |  |  | 6.8 (-4.25, 17.8) | 6.8 (-9.0, 22.7) | 5.3 (-9.6, 20.2) |
| **Iron deficiency (PF <15µg/L or TfR >8.3 mg/L)** | 53.7 | 60.6 | 59.5 | 59.9 |  |  |  |
| Model 1 |  |  |  |  | -0.3 (-12.4, 11.8) | -1.1 (-18.7, 16.5) | 0.32 (-16.4, 17.0) |
| Model 2 |  |  |  |  | 3.2 (-7.6, 14.0) | 2.0 (-13.7, 17.6) | 2.6 (-11.6, 16.9) |
| **IDA (anemia with PF <15µg/L or TfR >8.3 mg/L)** | 53.7 | 60.6 | 38.8 | 31.1 |  |  |  |
| Model 1 |  |  |  |  | 7.8 (-3.9, 19.5) | 2.2 (-14.7, 19.1) | 12.9 (-3.4, 29.1) |
| Model 2 |  |  |  |  | 10.4 (0.0, 20.7) * | 5.4(-10.3, 21.0) | 13.5 (0.1, 26.9) * |
| **VAD (RBP < 0.7µmol/L)** | 10.7 | 6.1 | 6.6 | 3.0 |  |  |  |
| Model 1 |  |  |  |  | 3.5 (-1.7, 8.7) | 5.6 (-4.8, 16.0) |  |
| Model 2 |  |  |  |  | 3.8 (-0.5, 8.1) | 7.0 (-1.6, 15.6) | -3.0 (-15.0, 8.9) |
| **Low or VAD (RBP < 1.05 µmol/L)** | 36.4 | 31.1 | 36.4 | 39.4 |  |  |  |
| Model 1 |  |  |  |  | -3.1 (-15.0, 8.7) | 1.3 (-16.5, 19.6) | -7.1 (-23.0, 8.7) |
| Model 2 |  |  |  |  | -3.9 (-14.8, 6.9) | 4.0 (-12.4, 20.4) | -9.2 (-23.7, 5.3) |

^1^All results are in percentages, reflecting the percentage point difference between the fortified compared to the unfortified biscuits group. Model 1 included the biscuits group and the study design effect (menarche status at enrolment). Model 2 adjusted for baseline micronutrient biomarkers (haemoglobin, PF, TfR and RBP) and the girl's baseline age, and HAZ; **P-value <* 0.05

**References**

1. WHO. Haemoglobin concentrations for the diagnosis of anaemia and assessment of severity. Geneva, Switzerland; 2011.

2. FAO and FANTA III. Minimum Dietary Diversity for Women: A Guide to Measurement. Food And Nutrition Technical Assistance Ⅲ. Rome, Italy; 2016.

3. FAO. The Food Insecurity Experience Scale ( FIES ). Guidance for translation : intended meanings of the questions and specific terms English Spanish Portuguese French Arabic Russian Chinese Albanian. Rome, Italy: FAO; 2015.

4. Filmer D, Pritchett LH. Estimating Wealth Effects Without Expenditure Data — or Tears : An application to educational enrollments in states of India. Demography. 2001;38(1):115–32.

5. Field A, Miles J. Discovering statistics using SAS. Los Angeles: Sage Publications; 2010.

6. Mei Z, Namaste SM, Serdula M, Suchdev PS, Rohner F, Flores-Ayala R, et al. Adjusting ferritin concentrations for inflammation: Biomarkers Reflecting Inflammation and Nutritional Determinants of Anemia (BRINDA) project. Am J Clin Nutr. 2017;106(Suppl):383S-389S.

7. Namaste SM, Aaron GJ, Varadhan R, Peerson JM, Suchdev PS. Methodologic approach for the Biomarkers Reflecting Inflammation and Nutritional Determinants of Anemia (BRINDA) project. Am J Clin Nutr. 2017;106:333S-347S.

8. Mei Z, Namaste SM, Serdula M, Suchdev PS, Rohner F, Flores-Ayala R, et al. Adjusting soluble transferrin receptor concentrations for inflammation: Biomarkers Reflecting Inflammation and Nutritional Determinants of Anemia (BRINDA) project. Am J Clin Nutr. 2017;106:383S-389S.
